# Prognostic value of cardiovascular magnetic resonance stress perfusion imaging in patients with atrial fibrillation

**DOI:** 10.1186/1532-429X-18-S1-P76

**Published:** 2016-01-27

**Authors:** Tamar Bigvava, Sarah B Nasser, Adelina Doltra, Bernhard Schnackenburg, Alexander Berger, Christoph Klein, Burkert Pieske, Rolf Gebker, Sebastian Kelle

**Affiliations:** 1 Cardiology, German Heart Institute Berlin, Berlin, Germany; 2 Tbilisi Heart and Vascular Clinic, Tbilisi, Georgia; 3 Dar Al Fouad Hospital, Cairo, Egypt; 4 Philips Healthcare, Hamburg, Germany

## Background

The purpose of this study was to assess the long-term prognostic value of cardiovascular magnetic resonance (CMR) stress perfusion in patients with atrial fibrillation who had suspected and known coronary artery disease (CAD) at initial stress CMR.

## Methods

130 consecutive patients with atrial fibrillation referred for perfusion stress CMR using either adenosine or regadenoson were followed for hard cardiovascular events defined as cardiac death or non-fatal myocardial infarction (MACE). Ischemia was defined as new onset of perfusion defects in at least two myocardial segments (positive test). Multivariable Cox regressions for MACE were performed to determine the prognostic value of CMR stress perfusion.

## Results

Hard cardiac events occurred in 4 (3.1%) patients during the follow-up period (mean: 21 ± 17 months). Patients without inducible perfusion defects (ischemia) experienced a substantially lower cumulative hard cardiovascular event rate (1%) than in patients with ischemia (9.1%) (p = 0.035) after 5 years (see Kaplan-Meier-curve).

## Conclusions

CMR stress perfusion in patients with atrial fibrillation can accurately identify patients, who are at increased risk for cardiac death and myocardial infarction, separating them from those with normal findings, who have a very low risk for future cardiac events.

**Figure 1:**
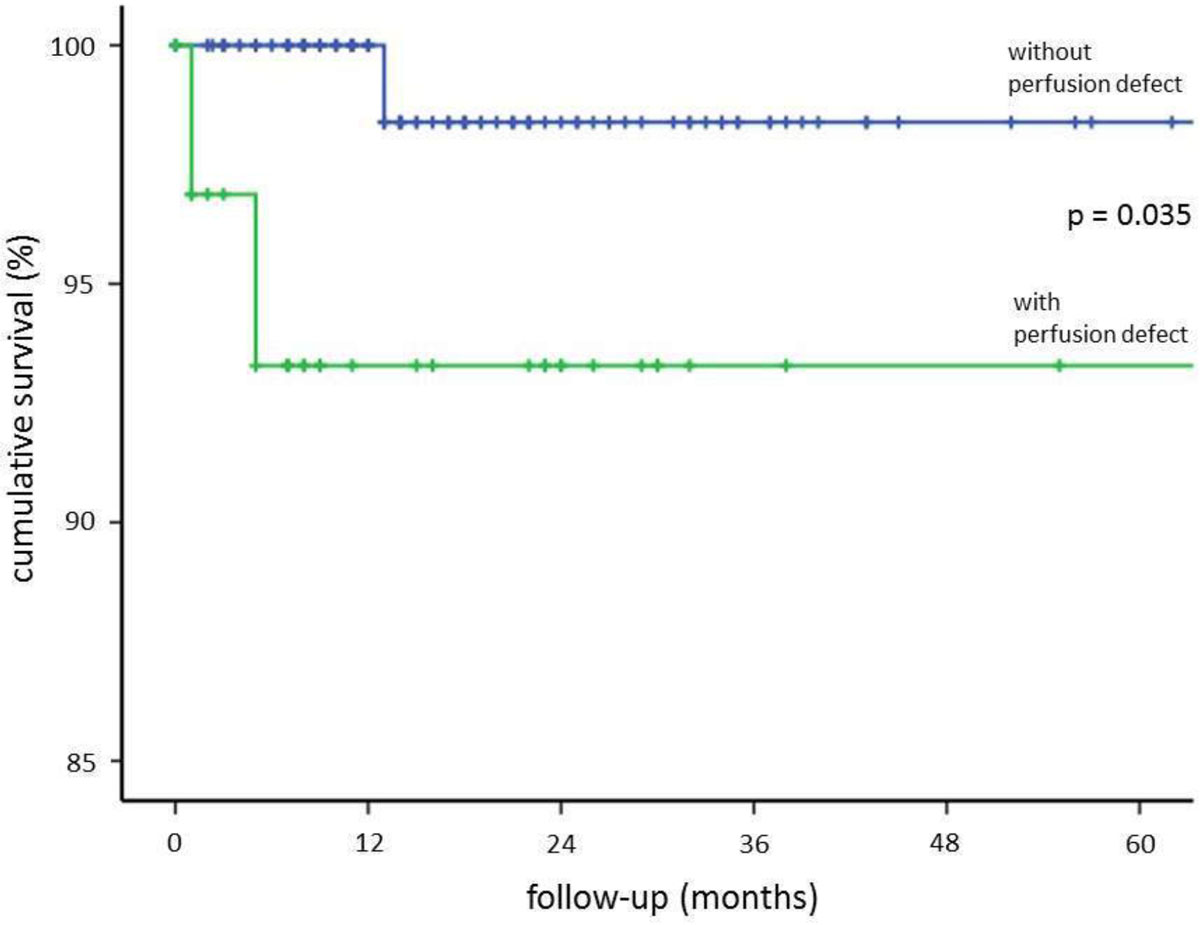
**Kaplan-Meier-curve demonstrating the prognostic value of stress perfusion CMR in 130 patients with atrial fibrillation**. Patients have been stratified dependent on the onset of inducible ischemia (perfusion defects).

